# Optimal use of granulocyte colony-stimulating factor prophylaxis to improve survival in cancer patients receiving treatment: An expert view

**DOI:** 10.1007/s00508-023-02300-6

**Published:** 2023-11-27

**Authors:** Pere Gascón, Ahmad Awada, Peeter Karihtala, Sylvie Lorenzen, Christoph Minichsdorfer

**Affiliations:** 1 Hospital Clinic, University of Barcelona, Barcelona, Spain; 2 Oncology Medicine Department, Institut Jules Bordet, Brussels, Belgium; 3 Department of Oncology, Helsinki University Hospital Comprehensive Cancer Center, University of Helsinki, Helsinki, Finland; 4 Technical University of Munich, Department of Hematology and Oncology, Klinikum rechts der Isar, Munich, Germany; 5 Division of Oncology, Department of Medicine I, Medical University of Vienna, Waehringer Guertel 18–20, 1090 Vienna, Austria

**Keywords:** Cancer supportive care, Chemotherapy dose intensity, Febrile neutropenia, G-CSF prophylaxis

## Abstract

**Background:**

Febrile neutropenia (FN) is a relatively common complication of cytotoxic chemotherapy. Prophylaxis with granulocyte colony-stimulating factor (G-CSF) can prevent FN and chemotherapy dose delays and enable the use of the higher dose intensities associated with a survival benefit; however, G‑CSF is not always used optimally.

**Summary:**

Five medical oncologists with a special interest in supportive care met to discuss the evidence for prophylaxis with G‑CSF to improve survival in cancer patients, identify reasons why this is not always done, and suggest potential solutions. The dose intensity of chemotherapy is critical for maximizing survival in cancer patients but may be reduced as a result of hematological toxicity, such as FN. Use of G‑CSF has been shown to increase the chances of achieving the planned dose intensity in various cancers, including early-stage breast cancer and non-Hodgkin lymphoma. All physicians treating cancer patients should consider the use of G‑CSF prophylaxis in patients receiving chemotherapy, paying particular attention to patient-related risk factors.

**Key messages:**

Strategies to optimize G‑CSF use include educating medical oncologists and pharmacists on the appropriate use of G‑CSF and informing patients about the efficacy of G‑CSF and its potential adverse effects. It is hoped that the evidence and opinions presented will help to encourage appropriate use of G‑CSF to support cancer patients at risk of FN in achieving the best possible outcomes from chemotherapy.

## Introduction

Febrile neutropenia (FN) is a relatively common and sometimes life-threatening complication of myelosuppressive cytotoxic chemotherapy [1]. The incidence and severity of FN vary according to the type of chemotherapy regimen used and are correlated with the intensity of chemotherapy [1, 2]. FN is defined in guidelines as an oral temperature of > 38.3 °C, or two readings of > 38.0 °C for 2 h, and an absolute neutrophil count (ANC) of < 0.5 × 10^9^/L or expected to fall to < 0.5 × 10^9^/L [1]. The Common Terminology Criteria for Adverse Events (CTCAE) defines FN as an ANC < 1000/mm^3^ with a single temperature of > 38.3 °C or a sustained temperature of ≥ 38 °C for > 1 h [3]. Potential consequences of FN include complicated life-threatening infections, prolonged hospitalization, chemotherapy dose delays, decreased relative dose intensities, decreased overall survival and progression-free survival, and increased costs to the healthcare system [1, 4–6]. For example, in a Danish study of 9018 patients treated with standard first-line chemotherapy for solid cancers, 845 (9.4%) experienced FN during the first-line treatment, and FN was associated with a significantly increased risk of all-cause mortality (incidence rate ratio, IRR, 1.39, 95% confidence interval, CI, 1.24–1.56), infectious mortality (IRR 1.94, 95% CI 1.43–2.62), and intensive care unit (ICU) admissions (IRR 2.28, 95% CI 1.60–3.24) [2]. In a US study of 1457 patients with metastatic cancer, more than 90% of FN episodes in patients not receiving prophylaxis with granulocyte colony-stimulating factor (G-CSF) required hospitalization [7]. All of these complications impose a financial burden.

In the pivotal phase III trial of primary prophylaxis with G‑CSF in 1991, the incidence rate of FN was 40% in patients who received G‑CSF compared with 77% in those who received placebo (*p* < 0.001) [8]. Consequently, between 1994 and 2000 the American Society of Clinical Oncology (ASCO) published guidelines recommending the use of G‑CSF in the first cycle of chemotherapy when the expected incidence rate of FN was ≥ 40% [9, 10]. Subsequently, G‑CSF prophylaxis was shown to reduce the incidence of FN by 92%, of FN-related hospitalization by 91%, and of FN-related use of anti-infective agents by 85% versus placebo in breast cancer patients who were receiving docetaxel and had an FN risk of 10–20% [11]. Guidelines published since that time have reflected this finding.

The current guidelines from ASCO, the European Society for Medical Oncology (ESMO), The European Organisation for Research and Treatment of Cancer (EORTC) and the National Comprehensive Cancer Network (NCCN) recommend primary prophylaxis with G‑CSF in patients receiving chemotherapy regimens associated with a high (≥ 20%) risk of FN and in those receiving chemotherapy regimens associated with an intermediate (10–20%) risk of FN who have additional risk factors, such as advanced age, comorbidities or history of previous FN [1, 12–14]. The latest NCCN guidelines state that G‑CSF is not routinely recommended for low-risk regimens (< 10%) but may be considered for patients with risk factors [12]. Primary prophylaxis is also recommended to support dose-dense or dose-intense chemotherapy strategies where these have a significant survival benefit [12–14]. Secondary G‑CSF prophylaxis is recommended for patients who have experienced an episode of FN in a previous cycle of chemotherapy or in the case of prolonged neutropenia or a dose-limiting neutropenic event [12–14]. During the COVID-19 pandemic, temporary changes were made to ESMO and ASCO guidelines to recommend G‑CSF prophylaxis in patients at intermediate risk (> 10%) [9, 15]. Chemotherapy regimens associated with a high or intermediate risk of FN are listed in the EORTC guidelines, along with important patient-related risk factors (Box 1) [13]. In addition to comorbidities and fragility in older patients, the decreased bone marrow reserve in older patients [16] makes age > 65 years a particularly important risk factor for FN.

### Box 1: Expert opinion—Most important patient-related risk factors to consider for G-CSF prophylaxis

- History of febrile neutropenia or neutropenia-related events.
- Age > 65 years.
- Fragility/frailty.
- Performance status, Eastern Cooperative Oncology Group (ECOG).
- Nutritional status and albumin level.
- Bone marrow involvement.
- Liver and kidney dysfunction.
- Other comorbidities, such as heart failure, heart disease or chronic obstructive pulmonary disease.

However, adherence to these guidelines is often suboptimal. In a retrospective US cohort study of 1457 patients with metastatic cancer, most patients for whom G‑CSF prophylaxis was recommended according to NCCN guidelines did not receive it. G‑CSF prophylaxis was used in 48.5% of patients on regimens carrying a high risk of FN (HR patients) and in only 13.9% of those on an intermediate-risk regimen with at least one additional risk factor (IR+1 patients) [7]. The FN incidence in cycle 1 among patients who did not receive G‑CSF was 7.8% for HR patients and 4.8% for IR+1 patients; FN incidences during the course were 16.9% and 15.9%, respectively. A study conducted in Germany in 2012 also reported suboptimal guideline adherence. A second representative study was conducted in 2015 to evaluate whether guideline implementation had improved, which included data from 573 patients with lung cancer and 801 patients with breast cancer. G‑CSF use in HR lung cancer patients increased from 15.4% to 47.8%, while the use in IR+1 patients increased from 38.8% to 44.3%. Adherence was higher in breast cancer patients, with G‑CSF use remaining steady in HR patients (85.6% in 2012 and 85.1% in 2015) and increasing in IR+1 patients (from 49.3% to 57.8%) [17].

This article considers the evidence for prophylaxis with G‑CSF to improve survival in cancer patients at risk of FN and examines some of the reasons why this is not always prescribed. It offers expert opinions on best practice for the use of G‑CSF and how the barriers to its use might be overcome.

## Use of G-CSF to support dose density and intensity and to improve survival

The dose intensity of chemotherapy is critical for maximizing survival in cancer patients, but may be reduced as a result of hematological toxicities, such as FN. For example, in a 20-year follow-up of 207 patients who received cyclophosphamide, methotrexate, and fluorouracil as adjuvant treatment for node-positive breast cancer in a clinical trial, both progression-free survival and overall survival were longer in patients who received ≥ 85% of the intended dose [18]. This was confirmed in a retrospective database study of 793 patients who received anthracycline-based non-taxane adjuvant chemotherapy for breast cancer. Disease-free survival and overall survival at 10 years were both affected by the number of delayed cycles (≤ 2 vs. > 2), the number of delayed days (< 15 vs. ≥ 15) and the relative dose intensity (RDI) (≥ 85% vs. < 85%) (all *p* < 0.05) [19]. Studies showed that using G‑CSF increases the chances of achieving ≥ 85% of planned dose intensity. For example, in a study of 407 breast cancer patients receiving adjuvant or neoadjuvant chemotherapy who had a neutropenic event, a target of 85% planned RDI was achieved in 75% of those randomized to receive G‑CSF compared with only 50% in the standard care arm (*p* < 0.0001) [20]. Thus, G‑CSF has a role in supporting dose intensity of chemotherapy regimens in (early-stage) breast cancer. The results of the recently published GIM 2 study showed improved long-term survival in patients receiving epirubicin, cyclophosphamide, and paclitaxel at 2‑week intervals with pegfilgrastim (*n* = 1002) versus 3‑week intervals without it (*n* = 1001) as adjuvant chemotherapy for node-positive breast cancer [21]. The estimated rate of 15-year disease-free survival was 61.1% (95% CI 57.5–64.5%) and 52.5% (95% CI 48.8–56.0%), respectively, in the dose-dense and control arms (hazard ratio, HR, 0.77, 95% CI 0.67–0.89; *p* = 0.0004), and the overall survival was also significantly longer (HR 0.72, 95% CI 0.60–0.86; *p* = 0.0004) [21].

Similar findings have been reported in non-Hodgkin lymphoma (NHL). In an evaluation of 210 Belgian patients with NHL treated with cyclophosphamide, doxorubicin, vincristine, and prednisone (CHOP) over 21 days (CHOP-21), those who received > 90% of the average relative dose intensity (ARDI) had significantly longer median survival than those who received ≤ 90% ARDI (*p* = 0.002) [22]. Hematological toxicity was the most common reason for receiving ≤ 90% ARDI [22]. Approximately 30% of these patients (a high proportion of whom were older and thus at risk of FN) and 23% of those in a smaller UK study received ARDI ≤ 90% and were therefore at risk of reduced survival [23]; however, dose-dense CHOP-14 chemotherapy supported with G‑CSF has been shown to be efficacious and well-tolerated in both young and older patients with NHL [24, 25]. Since the introduction of CHOP plus rituximab (R-CHOP), G‑CSF prophylaxis has been shown to enable on-time delivery of R‑CHOP-14 in patients with diffuse large B‑cell lymphoma, with ARDIs of 95% for doxorubicin and cyclophosphamide and 91% for vincristine, and low incidence of FN [26]; however, it should be borne in mind that rituximab has the potential to cause delayed and late-onset neutropenia that may vary in severity [27].

Additionally, dose density is very important in gastrointestinal cancer patients treated with curative intent. In the FLOT 4 trial, perioperative chemotherapy with fluorouracil plus leucovorin, oxaliplatin, and docetaxel (FLOT; *n* = 356) every 2 weeks improved overall survival in locally advanced, resectable gastric or gastroesophageal junction adenocarcinoma compared with fluorouracil or capecitabine plus cisplatin and epirubicin (ECF/ECX; *n* = 360) every 3 weeks [28]. Median overall survival was 50 months (95% CI 38 months–not reached) versus 35 months (95% CI 27–46 months) with a hazard ratio of 0.77 (95% CI 0.63–0.94); however, only 6% of patients in the ECF/ECX group and 5% in the FLOT group received G‑CSF prophylaxis with the first cycle; 21% in the ECF/ECX group and 34% in the FLOT group received it at any time during treatment. The incidence of grade ≥ 3 neutropenia was significantly higher with FLOT than with ECF/ECX (51% vs. 39%; *p* = 0.0017). Of note, 24% of patients were > 70 years of age with an increased risk of developing FN. As the rate of complicated neutropenia with FLOT is moderate, primary prophylaxis with G‑CSF is not required, although it is permitted. Consequently, in the phase II DANTE trial of FLOT plus atezolizumab versus FLOT alone in 295 patients with locally advanced, operable adenocarcinoma of the stomach or gastroesophageal junction, G‑CSF prophylaxis was recommended, usually from the second cycle, and 70–80% received it [29]. No difference in the number and severity of adverse events was seen with immunotherapy [30]. In the phase III DANTE trial, starting recruitment early in 2023, secondary prophylaxis with G‑CSF will be required for all subsequent cycles to avoid treatment delays, if one of the following criteria is applicable: occurrence of FN or infection in neutropenia at any time, occurrence of grade 4 neutropenia, delay of 1 therapy cycle by more than 3 days because of leukopenia or neutropenia.

Similar results have been obtained in other types of cancer. An analysis of data from three randomized controlled trials (RCT) for pancreatic adenocarcinoma showed that primary or secondary G‑CSF prophylaxis was associated with a higher rate of full dose intensity with FOLFIRINOX (odds ratio 5.07, 95% CI 1.52–16.90; *p* < 0.01) [31]. Full dose intensity was associated with a statistically nonsignificant increase in progression-free survival (HR 0.83, 95% CI 0.59–1.16; *p* = 0.27). A meta-analysis of 14 studies of primary G‑CSF prophylaxis in patients receiving chemotherapy for solid tumors or lymphomas showed that survival was significantly improved in patients receiving primary G‑CSF support compared with patients without primary G‑CSF support (relative risk, RR, for mortality 0.92, 95% CI 0.90–0.95; *p* < 0.0001) [32]. The largest improvement in survival was observed with dose-dense chemotherapy regimens with G‑CSF support, compared with controls receiving no G‑CSF support (RR 0.86, 95% CI 0.80–0.92; *p* < 0.0001).

## Best practice for G-CSF prophylaxis

It is important to pay particular attention to the many patients receiving intermediate-risk regimens, looking at comorbidities, nutritional status, and especially age > 65 years; any patient with one or more risk factors should receive G‑CSF starting 24 h after the first dose of chemotherapy [1, 12–14]. It is also important to bear in mind that patients seen in clinical practice are likely to be more sick and less fit than populations enrolled in clinical trials, so the risk of FN in real-world settings is likely to be higher and is likely to be underestimated. For example, a 2016 meta-analysis of 110 RCT cohorts and 65 observational cohorts (*n* = 50,069 patients in total) showed that an FN rate of 13% in clinical trials translated to a rate of 20% for real-world patients after risk factors such as age had been taken into account [33].

Box 2 provides the authors’ opinion on best practice for the use of G‑CSF.

### Box 2: Expert opinion—Best practice in G-CSF use

- All physicians treating cancer patients should consider the use of G‑CSF prophylaxis in patients receiving chemotherapy, paying particular attention to patient-related risk factors such as age > 65 years.
- G‑CSF prophylaxis is appropriate for all early-stage breast cancer patients receiving neoadjuvant or adjuvant chemotherapy, while those with metastatic disease should be assessed for risk factors.
  - G‑CSF prophylaxis should be used to maintain the higher dose intensities of chemotherapy that are associated with a survival benefit.
- 50–60% of gastrointestinal cancer patients treated with curative intent are candidates for prophylactic G‑CSF.
- G‑CSF prophylaxis is appropriate irrespective of whether the patient is receiving immunotherapy.
- There is a lot of variation in how G‑CSF is used, and patients may not be receiving the optimal duration of prophylaxis.
  - 5–6 days of short-acting G‑CSF may be sufficient in younger patients, whereas older patients are likely to need at least 7–8 days.
  - Treatment for longer than necessary can result in leukocytosis, and too short a treatment duration in high-risk patients can put their survival at risk.
- Long-acting and short-acting G‑CSF are equipotent in preventing FN episodes; however, long-acting G‑CSF compounds are only recommended for chemotherapy intervals of at least 14 days.
- Patients should be informed about the acute side effects of G‑CSF, especially bone pain.
  - Prophylactic prescription of an analgesic (e.g., nonsteroidal anti-inflammatory drugs) can be considered to improve treatment adherence.
- Steps should be taken to ensure that medical students, junior doctors, and pharmacists are educated about the importance of adhering to the guidelines on G‑CSF use, and that local protocols and electronic prescription tools are regularly updated.

## Overcoming barriers to optimal G-CSF use

As noted above, adherence to G‑CSF guidelines is often low; there may be various reasons for this. Adherence has been shown to vary depending on the type of cancer, the physician’s specialty, and the type of treatment center [17, 34, 35]. A survey including data from 573 patients with lung cancer and 801 patients with breast cancer (treated by 222 physicians) investigated G‑CSF guideline implementation in Germany [17]. The patterns that emerged indicated that hematologists/oncologists and gynecologists had significantly higher guideline adherence than pulmonologists, and that the former groups were more likely to adhere if they had more than 2 years of professional experience [17].

We believe that many less experienced physicians may not be aware of the impact that the availability of G‑CSF has had on the survival of cancer patients. Many physicians may underestimate the risk of hematological toxicities such as FN associated with different chemotherapy regimens and patient-related risk factors such as age. It is important to understand that G‑CSF prophylaxis is essential to enable patients to receive the full dose density they need to give them the best chance of survival in the context of (neo)adjuvant therapy. Senior physicians should ensure that less experienced colleagues treating cancer patients are educated about G‑CSF and other aspects of supportive care. We suggest that this is most efficiently done at a regional, local, or hospital level rather than at national or international meetings. The importance of G‑CSF prophylaxis should also be introduced to medical students as part of their training. Education should emphasize that supportive care not only improves the quality of life but also increases survival. The ESMO/ASCO recommendations for a global curriculum in medical oncology include understanding of treatment-related and patient-related risk factors for neutropenia and infections, understanding of the use of growth factor support, and understanding of the evaluation, prophylaxis, and treatment of FN in different patient populations [36]. There may be a case for revising the guidelines to make the decision easier for inexperienced physicians, leaving less to the individual’s judgement.

All physicians who treat cancer patients should be encouraged to consider whether the patient needs G‑CSF prophylaxis whenever they prescribe chemotherapy. Hospital protocols may include G‑CSF as well as other supportive care interventions that patients on chemotherapy may need, such as antiemetics, enabling routine prescription; however, this is usually only the case for high-risk regimens. For most patients who are receiving intermediate-risk regimens, physicians need to consider the risk factors for each patient. A US study examined the impact of implementing an electronic medical record (EMR) system on G‑CSF guideline adherence, showing an increase in adherence after implementation of the EMR (from 67.5% to 76.2%) [37]. The authors noted that adherence was much higher for HR patients (89.1%), for whom G‑CSF was added automatically to the order set, than for IR+1 patients (58.7%), in whom consideration of individual risk factors was needed. They suggested that the inclusion of an EMR-integrated decision-support tool to identify patient-specific risk factors and to notify the prescriber to consider G‑CSF prophylaxis would be likely to improve adherence in this group [37]. We note that newer therapies carrying an increased risk for FN might not be included in guidelines and might not be added to electronic prescription tools if these are not regularly updated. Local guidelines and protocols should be updated on a regular basis (e.g., annually). Furthermore, we note that pharmacists also have a role to play in ensuring that G‑CSF prophylaxis is prescribed in patients who need it; they should be involved, together with treating oncologists, in preparing protocols and treatment algorithms.

We believe that adverse events should not be a barrier to prescribing G‑CSF, as the benefit-risk balance is overwhelmingly in its favor. The most common adverse effects of G‑CSF are muscle and bone pain, headache, fatigue, and nausea [38–40]. Routine prescription of nonsteroidal anti-inflammatory drugs and paracetamol for patients receiving G‑CSF prophylaxis may be helpful in countering musculoskeletal pain and headache, and some patients with more severe bone pain may also need a mild opioid. Patient adherence to G‑CSF may be an issue if they are self-administering it at home. G‑CSF prophylaxis is most effective if started 24–48 h after chemotherapy [14], but some patients may use it at a different time or may not use it at all. For these reasons, informing and educating nurses and patients about the importance of G‑CSF and its potential adverse effects is crucial.

Two meta-analyses have shown an increased risk of secondary malignancies, including acute myeloid leukemia and myelodysplastic syndrome, in patients receiving G‑CSF prophylaxis (RR 1.92, 95% CI 1.19–3.07; *p* = 0.007 and 1.85, 95% CI 1.19–2.88; *p* < 0.01, respectively) [32, 41]; however, both showed reduced overall mortality risk with G‑CSF, and the increased risk of secondary malignancies may be primarily related to an increased dose of chemotherapeutic agents with known leukemogenic potential [32, 41].

Finally, with the wide availability and proven cost-effectiveness of biosimilars [42–44], cost should no longer be a barrier to the use of G‑CSF prophylaxis.

## Conclusion

FN and neutropenia-related events such as infections are relatively common complications of cytotoxic chemotherapy that can compromise the survival of cancer patients. G‑CSF prophylaxis is important in preventing FN and dose delays or reductions, as well as enabling the use of higher dose intensities of (neo)adjuvant chemotherapy that are associated with a survival benefit. All physicians treating cancer patients should consider the use of G‑CSF prophylaxis in patients receiving chemotherapy, paying particular attention to patient-related risk factors such as age > 65 years. Education of medical students, doctors, nurses, pharmacists, and patients is essential to improve awareness of the risks of FN and to ensure appropriate use of G‑CSF prophylaxis. An update of the 2016 ESMO guidelines on the management of FN would be welcomed.
